# Leveraging machine learning-based approaches to assess human papillomavirus vaccination sentiment trends with Twitter data

**DOI:** 10.1186/s12911-017-0469-6

**Published:** 2017-07-05

**Authors:** Jingcheng Du, Jun Xu, Hsing-Yi Song, Cui Tao

**Affiliations:** University of Texas School of Biomedical Informatics, 7000 Fannin St Suite 600, Houston, TX 77030 USA

**Keywords:** Sentiment analysis, Human papillomavirus vaccines, Machine learning, Twitter, Hierarchical classification

## Abstract

**Background:**

As one of the serious public health issues, vaccination refusal has been attracting more and more attention, especially for newly approved human papillomavirus (HPV) vaccines. Understanding public opinion towards HPV vaccines, especially concerns on social media, is of significant importance for HPV vaccination promotion.

**Methods:**

In this study, we leveraged a hierarchical machine learning based sentiment analysis system to extract public opinions towards HPV vaccines from Twitter. English tweets containing HPV vaccines-related keywords were collected from November 2, 2015 to March 28, 2016. Manual annotation was done to evaluate the performance of the system on the unannotated tweets corpus. Followed time series analysis was applied to this corpus to track the trends of machine-deduced sentiments and their associations with different days of the week.

**Results:**

The evaluation of the unannotated tweets corpus showed that the micro-averaging F scores have reached 0.786. The learning system deduced the sentiment labels for 184,214 tweets in the collected unannotated tweets corpus. Time series analysis identified a coincidence between mainstream outcome and Twitter contents. A weak trend was found for “Negative” tweets that decreased firstly and began to increase later; an opposite trend was identified for “Positive” tweets. Tweets that contain the worries on efficacy for HPV vaccines showed a relative significant decreasing trend. Strong associations were found between some sentiments (“Positive”, “Negative”, “Negative-Safety” and “Negative-Others”) with different days of the week.

**Conclusions:**

Our efforts on sentiment analysis for newly approved HPV vaccines provide us an automatic and instant way to extract public opinion and understand the concerns on Twitter. Our approaches can provide a feedback to public health professionals to monitor online public response, examine the effectiveness of their HPV vaccination promotion strategies and adjust their promotion plans.

**Electronic supplementary material:**

The online version of this article (doi:10.1186/s12911-017-0469-6) contains supplementary material, which is available to authorized users.

## Background

With the rise of social media and the burgeoning volume of user-generated data, there is a growing interest in using social media data for public health related studies. Social media surveillance has proven its value for many public health issues, such as estimating of epidemiological patterns [1], forecasting disease outbreak [2, 3], detecting drug adverse effects [4], and assessing vaccination [5, 6]. With 310 million monthly active users and 500 million tweets posting per day [7], Twitter is one of the largest and most popular social media in US and in the world. The length limit of 140 characters per tweet also makes users post more concisely and more expressively than other social networks and blogs [8]. These characteristics make Twitter a very valuable data source for public health informatics researchers.

Vaccination refusal has been a serious issue for human papillomavirus (HPV) vaccines [9]. Introduced in 2006, HPV vaccine can be used to prevent most cancers caused by HPV infections. However, compared to other recommended vaccines, HPV vaccines coverage in USA is still quite low especially among adolescents [10]. Anti-vaccine rhetoric directed at HPV vaccines in media and online appears to be able to alter vaccine acceptance and decision-making [5]. As individuals’ decisions about whether or not to immunize are not usually made rationally nor at one moment in time [11] and individual health behaviors appear to be modulated by opinions from social networks [6], understanding public opinion towards HPV vaccines in social media is of significant importance for HPV vaccination promotion. Moreover, the surveillance of real-time Twitter information flow could provide timely updates when scares arise, and instant feedback to public health agencies to examine and adjust their strategies to improve future HPV vaccines uptake and adherence.

Unlike traditional surveying methods that are labor-intensive and expensive [6], we propose to leverage machine learning approaches to extract public opinion from tweets automatically. This is called sentiment analysis (SA) in the field of natural language processing (NLP). In our previous work, we developed a machine learning based sentiment analysis system that can hierarchically classify HPV vaccine-related tweets into 10 categories [12]. This system not only will deduce the sentiment polarity of a tweet at the high level (e.g., “Positive”, “Negative” and “Neutral”), but will also further identify the exact reasononing behind a negative opinion (e.g., “Safety”, “Efficacy”, etc.). In this study, we evaluated this system on a large-scale unannotated tweets corpus and deduce the sentiment labels of those tweets. Additional time series analysis and regression models were applied to track the changes and to identify the patterns of different sentiments toward HPV vaccines over time.

## Methods

### Data resource

We used a set of keywords (hpv, human papillomavirus, gardasil, and cervarix) to collect English tweets by using Twitter Streaming APIs. The tweets corpus was collected from November 2, 2015 to March 28, 2016. There were 184,214 tweets collected during that time period. We will use this corpus to evaluate the sentiment analysis system developed in our previous work and leverage the system to extract and analyze public opinion toward HPV vaccines from the unannotated tweets corpus.

### Machine learning system

**Fig. 1:**
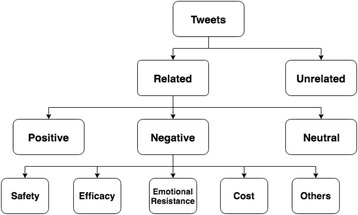
Sentiment classification scheme for HPV vaccine related tweets [12]

**Fig. 2:**
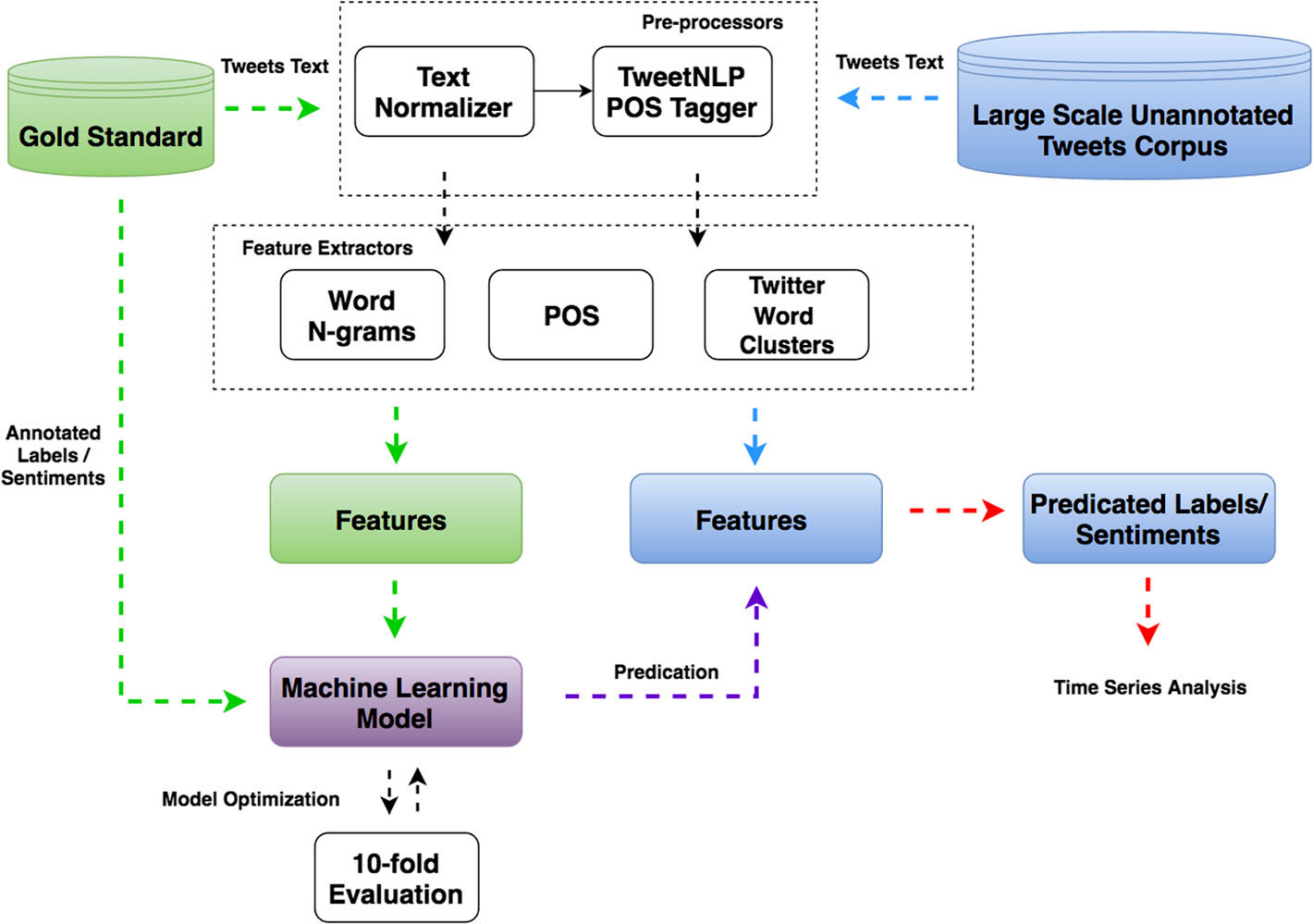
Overview of the machine learning based system for tweets sentiment analysis

A machine learning based sentiment analysis system was developed in our previous work to extract public opinions from HPV vaccines related tweets [12]. This system was able to classify tweets into multiple sentiment categories. Figure 1 shows the sentiment classification scheme for the HPV vaccines related tweets. Detailed definitions of each category were provided in Additional file 1. By leveraging hierarchical classification methods (three SVM models) with optimized feature sets and model parameters, this system has achieved the micro-averaging F score at 0.7442 on the gold standard [12]. The overview of the sentiment analysis system can be seen in Fig. 2.

### Evaluation of the system on the unannotated tweets corpus

To evaluate system performance on the unannotated tweets corpus, we randomly selected 500 tweets from the unannotated tweets corpus and manually annotated those tweets according to the sentiment classification scheme. Then, the machine learning system was applied to deduce the sentiment labels of these annotated tweets. Machine learning system- deduced sentiment labels were compared with manually annotated labels. Standard metrics including precision, recall and F measure were then calculated to evaluate the performance of the system. We also calculated the micro-averaged and macro-averaged score to evaluate the overall performance on all sentiment categories. To calculate the micro-averaged score, we summed up all the individual true positives, false positives, and false negatives. For the macro-averaged score, we calculated the average score of the F measure of all sentiment categories.

### Time series analysis on predicated sentiments

After the evaluation, we leveraged our system to deduce the sentiment labels for all the tweets in the unannotated tweets corpus. Longitudinal deduced counts and rates for different sentiment categories were calculated. Time series trends for different sentiment categories were graphed and analyzed by Tableau. As social media posting behavior is associated with different days of the week [13, 14], we also calculated the averaged rates of different sentiment groups for different days of the week. Linear and quadratic regression models were used to fit the data to explore trends and associations.

## Results

### Machine learning system evaluation results

Among the 500 randomly selected tweets from the large corpus, our manual annotation found that 193 of them were “Unrelated” to HPV vaccine sentiments, 116 tweets were “Neutral”, and 106 tweets were “Positive”. There were also 65 tweets and 20 tweets that were annotated into “NegSafety” and “NegOthers” sentiment categories, respectively. The micro-averaged and macro-averaged of F scores have reached 0.786 and 0.708 respectively. The overall performance was promising. The detailed evaluation results for the overall performance and for each category can be seen in Table 1.

**Table 1:** Machine learning system evaluation on 500 randomly selected tweets from the unannotated tweets corpus

Category				Precision	Recall	F measure
Overall	Micro-averaging			0.7860	0.7860	0.7860
	Macro-averaging			0.7112	0.7051	0.7081
Per Category	Unrelated			0.9337	0.9482	0.9409
	Related	Positive		0.6596	0.8774	0.7530
		Neutral		0.7586	0.5690	0.6502
		Negative	Safety	0.8039	0.6308	0.7069
			Others	0.4000	0.5000	0.4444

### Overall description of the predicated sentiments

**Fig. 3:**
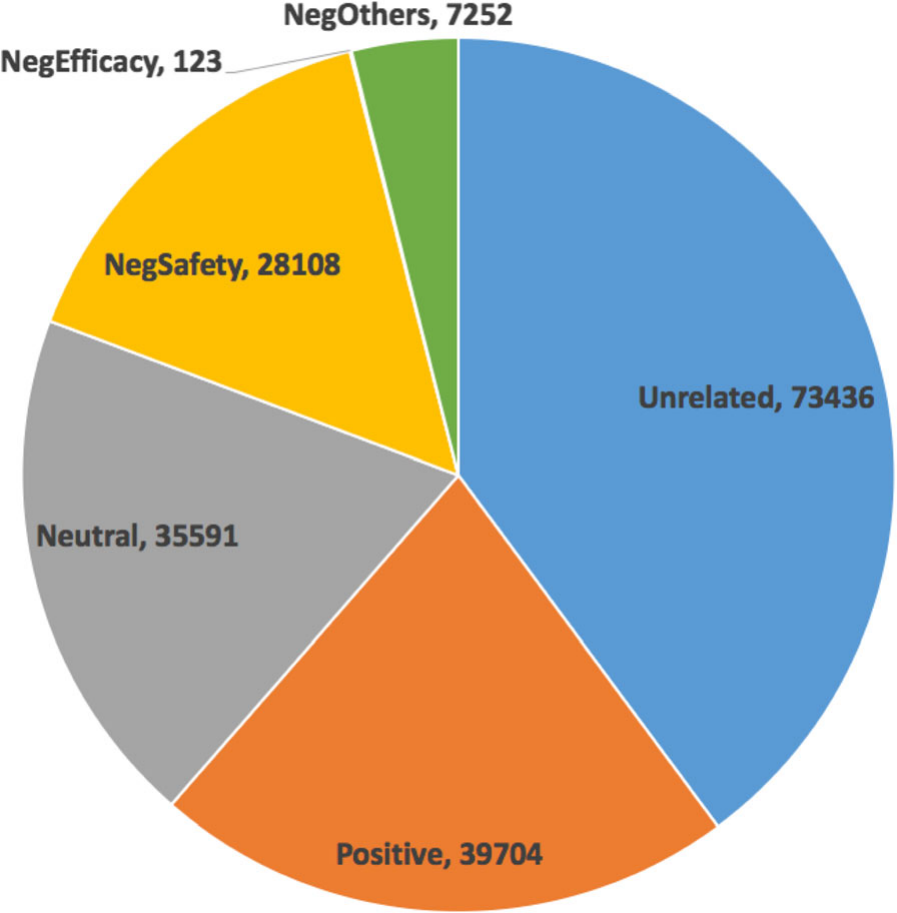
Sentiments distribution in large scale unannotated HPV vaccines related tweets corpus. (Neg: Negative)

Our machine learning system then deduced the sentiment labels for 184,214 tweets in the large unannotated tweets corpus from the study period. Overall, 110,778 (60.13%) tweets were classified into the “Related” group. Among the related tweets, 39,704 (35.8%) of them showed positive opinions; 35,591 (32.1%) tweets are categorized into “Neutral”; and 35,482 (32.0%) tweets were categorized into “Negative”. The largest group under the “Negative” tweets is “NegSafety”. There were 28,108 tweets classified into this category. Besides, 7252 tweets and 123 tweets were categorized into “NegOthers” and “NegEfficacy” respectively. No tweets were classified into “NegCost” or “NegResistant”. Detailed sentiments distribution can be seen in Fig. 3. Sample tweets predicated by the machine learning system for different sentiment categories were provided in the Additional file 2.

### Time series analysis for different sentiment categories

**Fig. 4:**
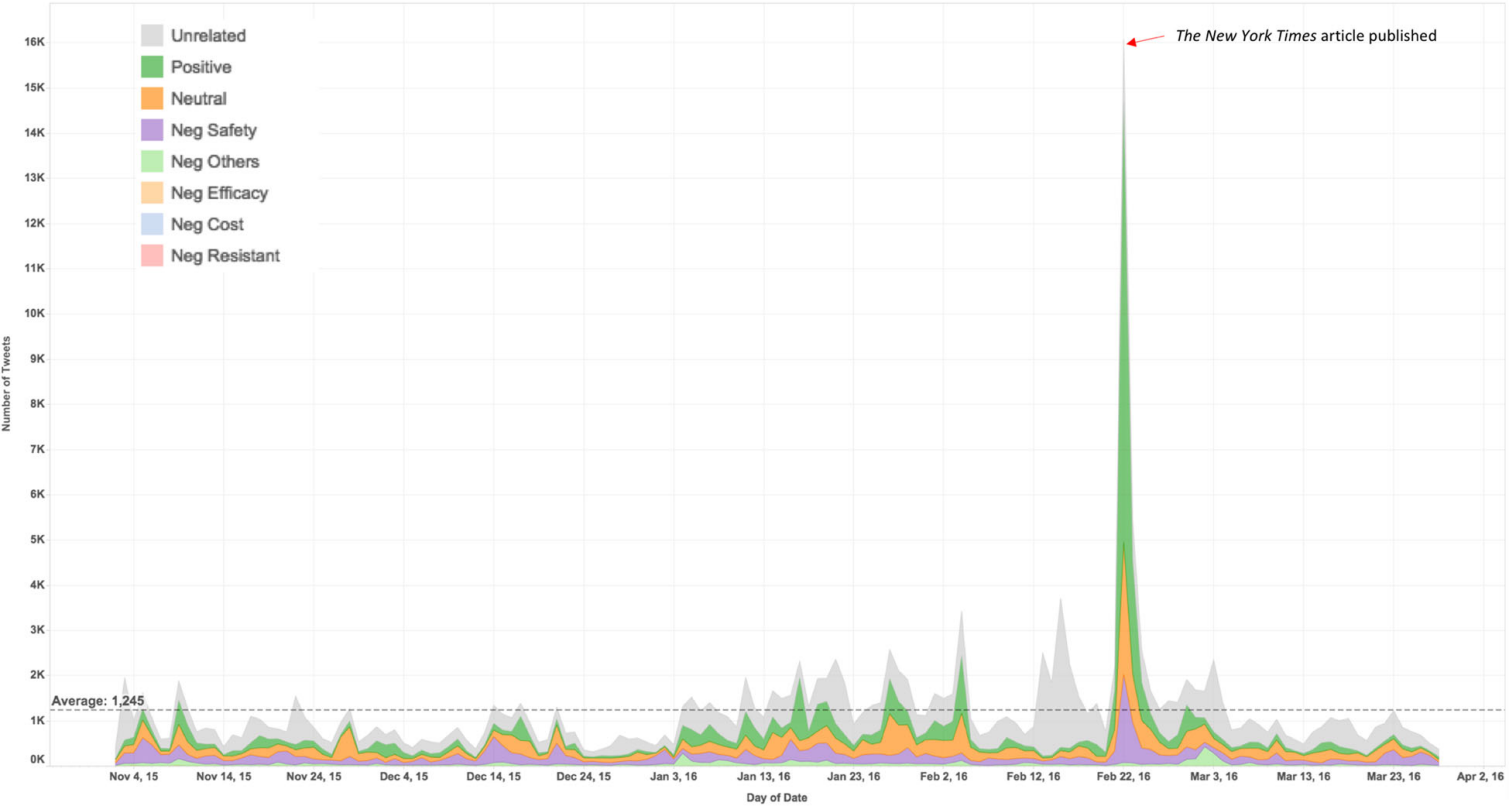
Stacked line chart for the number of tweets containing different sentiments from November 2, 2015 to March 28, 2016

Figure 4 presents an overview of the changing number of different sentiment tweets from November 2, 2015 to March 28, 2016. The average posts for all categories were 1245 per day. A sharp peak was found on Feb 22, 2016. This peak was coincided with an article on *The New York Times* titled “HPV Sharply Reduced in Teenage Girls Following Vaccine, Study Says” [15]. This article was published exactly on Feb 22, 2016. This coincidence showed an interaction between mainstream events and Twitter contents.

**Fig. 5:**
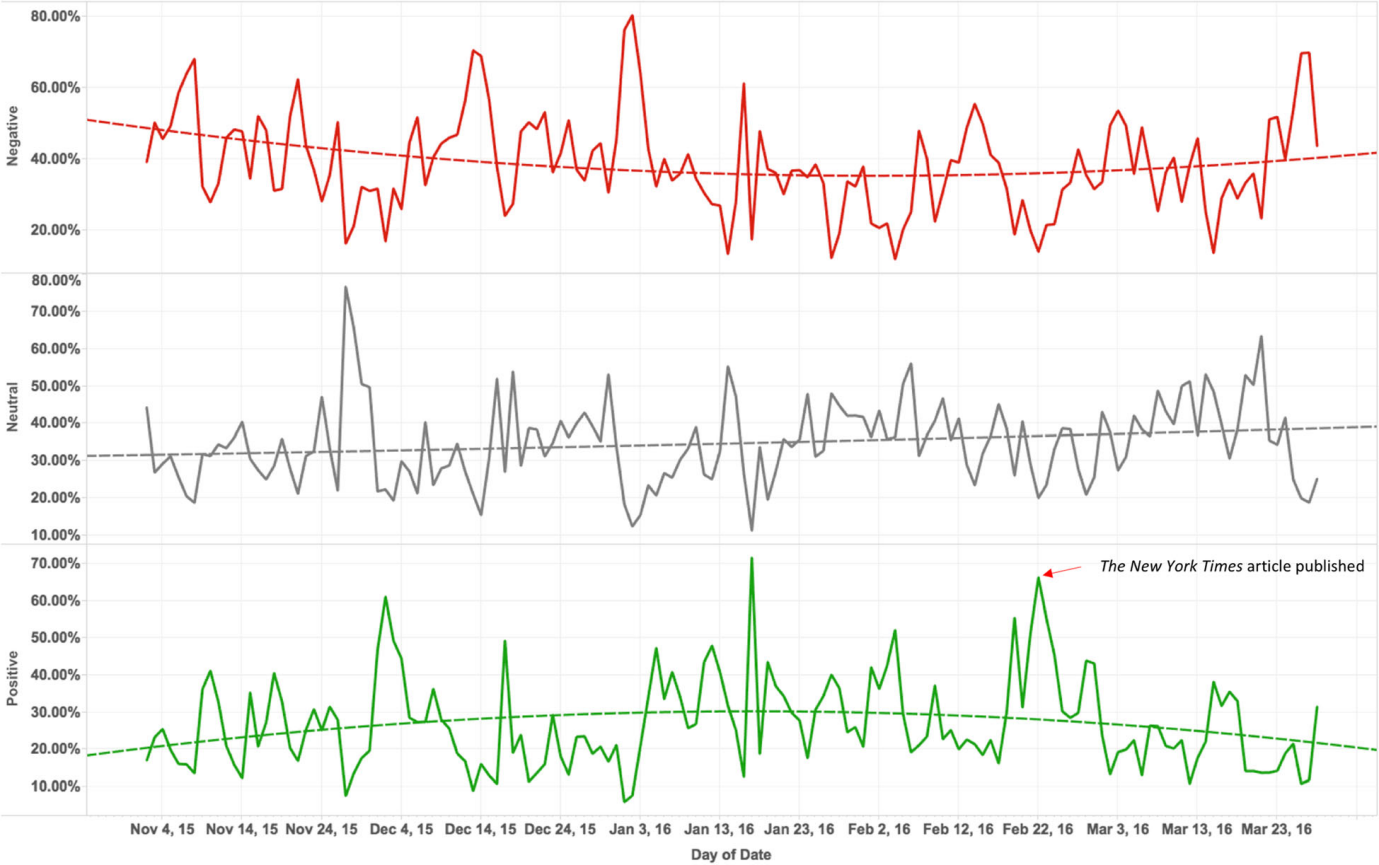
The relative proportions of tweets containing Negative, Neutral and Positive opinions

We calculated the relative daily proportion of “Positive”, “Negative” and “Neutral” tweets to “Related” tweets respectively. Figure 5 shows the time series of different sentiments proportion from November 2, 2015 to March 28, 2016. One of the peaks for “Positive” was found around Feb 22, 2016 (at 66.21%), when *The New York Times* published the aforementioned article. It can be used as an example to show how real-world media can influence the HPV vaccines public opinion on social media.

Quadratic models were then used to fit the time series data to explore trends. We can observe that there were strong fluctuations of the relative proportion for these three sentiment groups. For the “Negative” group, we can see a weak trend that the relative proportion decreased firstly and began to increase around Feb, 2016; for the “Positive” group, we can observe a trend opposite to the “Negative” group, which increased at first and started to decrease around Feb, 2016; for “Neutral” tweets, the curve of the fitted quadratic model was relatively flat.

**Fig. 6:**
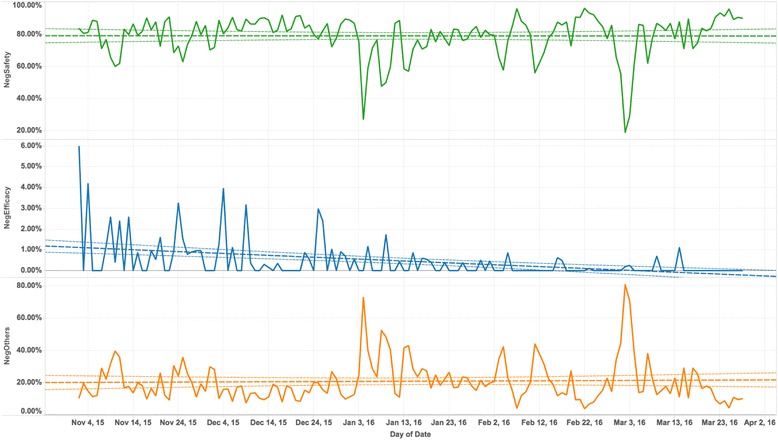
The relative proportions of “NegSafety”, “NegEfficacy” and “NegOthers” tweets to “Negative” tweets

To explore the time series trend of the sub-category of “Negative”, we further calculated the relative daily proportion of “NegSafety”, “NegEfficacy” and “NegOthers” tweets to “Negative” tweets respectively, see Fig. 6, Two main peaks have been found for “NegOthers” sentiment group: 72.92% on Jan 4, 2016 and 80.95% on Mar 2, 2016. Linear models were fitted to explore the trends. We can observe that there was relative significant decreasing trend for “NegEfficacy” sentiment group. For “NegSafety” and “NegOthers” groups, no significant trends were identified.

**Fig. 7:**
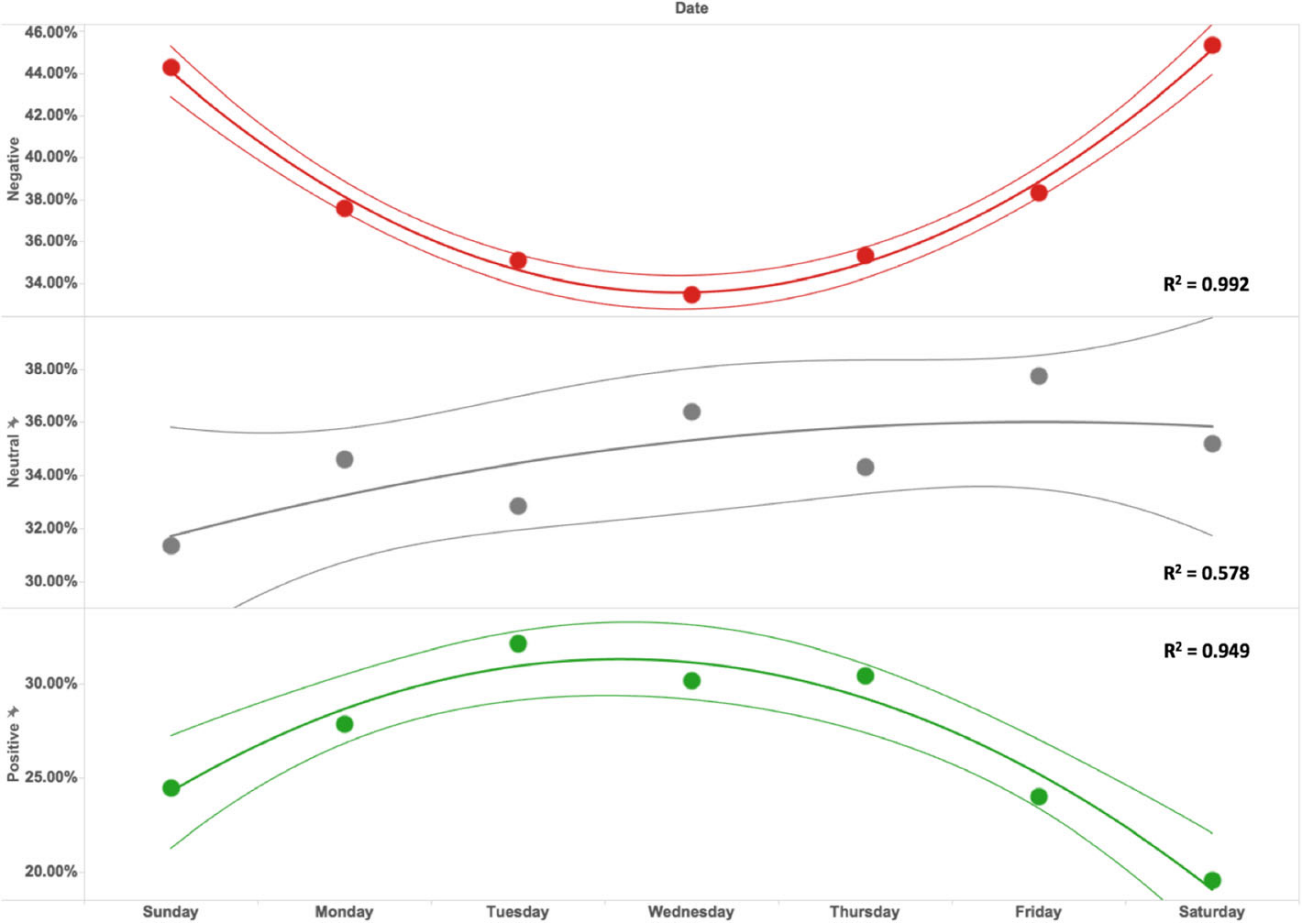
The association of different days of the week with the relative proportions of tweets containing Negative, Neutral and Positive opinions

In order to explore the association of people’s posting behavior and sentiments with different days of the week, we went one step further and calculated the average rates of different sentiment groups on different days of the week. We applied the quadratic models to fit the data. Figure 7 shows the association of “Negative”, “Neutral” and “Positive” sentiment groups with different days of the week. For the “Negative” sentiment group, we found that the quadratic models fit the data quite well (R^2^ = 0.992). The average rate for “Negative” came to the bottom (around 34%) on Wednesdays and reached the peak on weekends. For “Positive” tweets, the quadratic model also fitted the trends quite well (R^2^ = 0.992). The trend for “Positive” tweets is quite opposite to “Negative” tweets. The average rate for “Positive” reached the peak at the middle of a week and came to the bottom on the weekends. No significant association was found for the “Neutral” sentiment group.

**Fig. 8:**
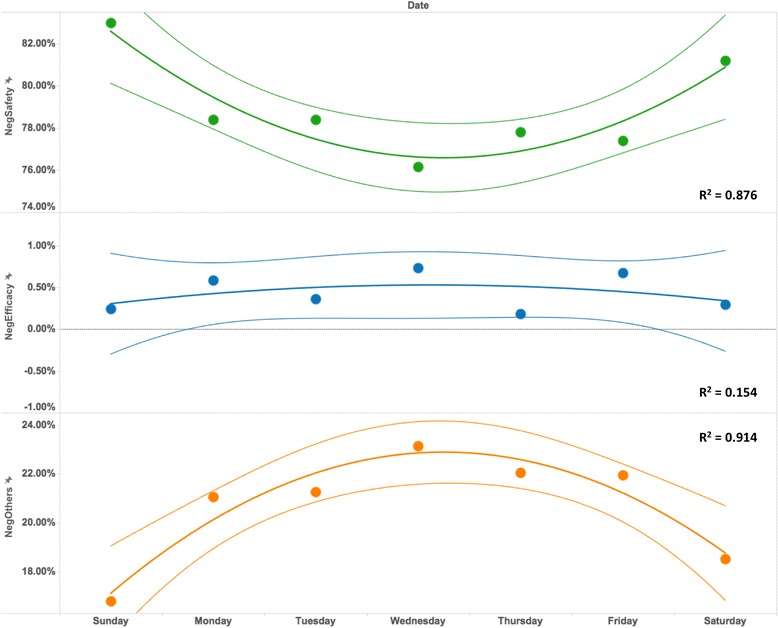
The association of different days of the week with the relative proportions of “NegSafety”, “NegEfficacy” and “NegOthers” tweets

The association of “NegSafety”, “NegEfficacy”, and “NegOthers” tweets with different days of the week can be seen in Fig. 8. The quadratic models fitted the “NegSafety” and “NegOthers” sentiment groups quite well (R^2^ at 0.876 and 0.917 respectively). The relative proportion for “NegSafety” reached the bottom (around 76%) on Wednesdays and peaked on weekends. For “NegOthers” tweets, the average rate reached the peak at the middle of the week and was lowest on the weekends. No significant association was found for the “NegEfficacy” sentiment group.

## Discussion

Our analysis found the coincidence between mainstream events (*The New York Times* article) and Twitter contents. We also found that the activity on mainstream media can have a significant influence on HPV vaccine public opinion on Twitter (with 66.21% positive rate on the article posting day compared to 35.8% positive rate for all the tweets). It is safe to say that Twitter is not an isolated world and that promotion efforts in mainstream media could have a positive impact on public opinion on Twitter.

Further analysis helped us find more trends and patterns for different sentiments on HPV vaccines. We observed a weak trend for “Negative” tweets that decreased firstly and began to increase later; an opposite trend was identified for “Positive” tweets. We also found the tweets that contain the concerns about efficacy for HPV vaccines showed a relative significant decreasing trend. Strong associations were found between different sentiments and different days of a week. Average rates for the “Negative” tweets reached to the bottom on Wednesdays and reached the peak on weekends; an opposite association was found for “Positive” tweets. For the sub-categories of the “Negative” sentiment group, strong associations between the numbers of tweets and days of a week were also found for “NegSafety” and “NegOthers”.

One third of parents are distrustful of newer vaccines [11]. Our efforts on sentiment analysis for newly approved HPV vaccines provide an automatic and instant way to extract public opinion and understand the concerns using Twitter data. Our system can provide feedback to public health professionals to monitor online public response and examine the effectiveness of their HPV vaccine promotion strategies. The associations found for different sentiments with different days of week could also be very helpful for public health professionals in adjusting their promotion plans. For example, they can deliver more persuasive messages on weekends instead of the middle of the week, as the negative opinions are more prevalent on these days.

A significant issue for the machine learning system is the un-satisfactory performance on the minority categories. However, as we observed that the proportion of those tweets is very small, this will not significantly influence the results. Another limitation of our approach is study population bias. As the Twitter population is not representative of the general population, the public opinions on Twitter cannot be used to fully represent the opinions of the general public. However, previous study showed the correlation between sentiments expressed online and CDC-estimated vaccination rates [6], and therefore we believe our results are meaningful and can be used to be assess general public opinion.

## Conclusion

In order to understand public opinion about HPV vaccines, we leveraged the machine learning-based sentiment analysis system to automatically extract public opinion towards HPV vaccine from a large unannotated tweets corpus that contains HPV vaccines related keywords. The evaluation of the system on the large tweets corpus was promising, with micro-averaged score at 0.786 and macro-averaged score at 0.7081 respectively on the sampling dataset. Further time series analysis was done and identified trends and patterns of different sentiments and their association with different days of the week. Our findings can be provided to the health professionals to propose more precise and efficient plan to resolve public concerns on Twitter and come up with promotion plan to increase HPV vaccine uptake finally.

## Additional files

Additional file 1: Table S1. Detailed definitions of different sentiment categories for HPV vaccine related tweets. (DOC 30 kb)

Additional file 2: Table S2. Sample tweets predicated by the machine learning system. URLs and Twitter user names have been removed. (DOC 30 kb)
