# Supplementary material for: Leveraging machine learning-based approaches to assess human papillomavirus vaccination sentiment trends with Twitter data
Source: BMC Med Inform Decis Mak. 2017 Jul 5;17(Suppl 2):69. doi: 10.1186/s12911-017-0469-6 (PMC5506590; doi:10.1186/s12911-017-0469-6)
Supplement: Supplementary file 1 — Detailed definitions of different sentiment categories for HPV vaccine related tweets. (DOC 30 kb) [file 12911_2017_469_MOESM1_ESM.doc]

**Additional file 1**

**Table S1** Detailed definitions of different sentiment categories for HPV vaccine related tweets

| **Sentiment** | | **Description** |
| --- | --- | --- |
| Positive | | Show positive opinion or prompt the uptake of HPV vaccine |
| Negative | Safety | Concerns or doubt on the safety issues of HPV vaccine or present vaccine injuries |
|  | Efficacy | Concerns or doubt on the effectiveness over HPV vaccine |
|  | Cost | Concerns on the cost of HPV vaccine (e.g.: money or time) |
|  | Resistant | Resistance to HPV vaccines due to cultural or emotional issues |
|  | Others | Other concerns |
| Neutral | | Related to HPV vaccine topic but contains no sentiment or sentiment is unclear or contains both negative and positive sentiment |
| Unrelated | | Not related to HPV vaccine topic |
