# Supplementary material for: Leveraging machine learning-based approaches to assess human papillomavirus vaccination sentiment trends with Twitter data
Source: BMC Med Inform Decis Mak. 2017 Jul 5;17(Suppl 2):69. doi: 10.1186/s12911-017-0469-6 (PMC5506590; doi:10.1186/s12911-017-0469-6)
Supplement: Supplementary file 2 — Sample tweets predicated by the machine learning system. URLs and Twitter user names have been removed. (DOC 30 kb) [file 12911_2017_469_MOESM2_ESM.doc]

**Additional File 2**

**Table S2** Sample tweets predicated by the machine learning system. URLs and Twitter user names have been removed

| **Sentiment** | | **Sample Tweets** |
| --- | --- | --- |
| Positive | | “Thanks to the mom who chose to help protect her kids from cancer by starting the HPV vaccine series today!”  “We know it's effective. So why is there opposition to the HPV vaccine ?” |
| Negative | Safety | “#Gardasil wreaking more havoc by destroying #ovaries of young girls: #cancer”  “Hundreds of vaccines pushed by the NHS. Scandal of hundreds of thousands girls getting ill from HPV vaccine made...” |
|  | Efficacy | “There is no proof #gardasil has or will prevent cancer. Virus? Maybe. Cancer? No.”  “Utah health official bans Gardasil, says Merck exaggerated benefits and FDA approved too quickly” |
|  | Others | “This Might Make You Think Twice About Injecting Your Child With Gardasil”  “I have a friend who IDs as asexual, who isn't aromantic who lets her doctor give her the HPV shot not because she goes” |
| Neutral | | “Cancer centers alarmed over low number of HPV vaccinations”  “Numbers game: Researchers explore whether fewer than 3 doses of HPV vaccine are effective” |
| Unrelated | | “CD56-positive lymphocyte infiltration in relation to human papillomavirus association and prognostic significance”  “Oh dear, oh dear, oh dear.” |
